# Quantum dots incorporated magnetic nanoparticles for imaging colon carcinoma cells

**DOI:** 10.1186/1477-3155-11-28

**Published:** 2013-08-17

**Authors:** Syed Rahin Ahmed, Jinhua Dong, Megumi Yui, Tatsuya Kato, Jaebeom Lee, Enoch Y Park

**Affiliations:** 1 Department of Bioscience, Graduate School of Science and Technology, Shizuoka University, 836 Ohya Suruga- ku, Shizuoka 422-8529, Japan; 2 Department of Nano Fusion Technology, College of Nanoscience and Nanotechnology, Pusan National University, Miryang 627-706, Korea; 3 Department of Applied Biological Chemistry, Shizuoka University, 836 Ohya Suruga- ku, Shizuoka 422-8529, Japan; 4 Green Chemistry Research Division, Research Institute of Green Science and Technology, Shizuoka University, 836 Ohya Suruga- ku, Shizuoka 422-8529, Japan; 5 Department of Cogno-Mechatronics Engineering, Pusan National University, Busan 609-735, Korea

**Keywords:** Core-shell structure, Magnetic nanoparticles, Quantum dots, Fluorescent nanoparticles, Cell imaging

## Abstract

**Background:**

Engineered multifunctional nanoparticles (NPs) have made a tremendous impact on the biomedical sciences, with advances in imaging, sensing and bioseparation. In particular, the combination of optical and magnetic responses through a single particle system allows us to serve as novel multimodal molecular imaging contrast agents in clinical settings. Despite of essential medical imaging modalities and of significant clinical application, only few nanocomposites have been developed with dual imaging contrast. A new method for preparing quantum dots (QDs) incorporated magnetic nanoparticles (MNPs) based on layer-by-layer (LbL) self-assembly techniques have developed and used for cancer cells imaging.

**Methods:**

Here, citrate - capped negatively charged Fe_3_O_4_ NPs were prepared and coated with positively - charged hexadecyltrimethyl ammonium bromide (CTAB). Then, thiol - capped negatively charged CdTe QDs were electrostatically bound with CTAB. Morphological, optical and magnetic properties of the fluorescent magnetic nanoparticles (FMNPs) were characterized. Prepared FMNPs were additionally conjugated with hCC49 antibodies fragment antigen binding (Fab) having binding affinity to sialylated sugar chain of TAG-72 region of LS174T cancer cells, which was prepared silkworm expression system, and then were used for imaging colon carcinoma cells.

**Results:**

The prepared nanocomposites were magnetically responsive and fluorescent, simultaneously that are useful for efficient cellular imaging, optical sensing and magnetic separation. Transmission electron microscopy (TEM) and dynamic light scattering (DLS) revealed that the particle size is around 50 nm in diameter with inner magnetic core and outer CdTe QDs core-shell structure. Cytotoxicity test of prepared FMNPs indicates high viability in Vero cells. NPs conjugated with anti cancer antibodies were successfully labeled on colon carcinoma cells (LS174) *in vitro* and showed significant specificity to target cells.

**Conclusion:**

The present report demonstrates a simple synthesis of CdTe QDs-Fe_3_O_4_ NPs. The surface of the prepared FMNPs was enabled simple conjugation to monoclonal antibodies by electrostatic interaction. This property further extended their *in vitro* applications as cellular imaging contrast agents. Such labeling of cells with new fluorescent-magneto nanoprobes for living detection is of interest to various biomedical applications and has demonstrated the potential for future medical use.

## Background

The merging of dual natured components of optical and magnetic properties on nanoscale regime can enable new advance in molecular imaging and medical theranosis that are essential for early detection and rapid treatment of diseases [1,2]. Magnetic nanoparticles (MNPs), e.g. Fe_3_O_4_ NPs, are of immense importance in the emerging area of nanomedicine because of their ability to be manipulated and detected with an external magnetic field. They can be used in numerous applications, both *in vitro* and *in vivo*, such as magnetic resonance imaging (MRI) contrast enhancement [3], bioseparation [4], biosensing [5], cancer therapy using magnetic hyperthermia [6], and targeted drug delivery [7]. Although MNPs appear to be the currently preferred cell-labeling materials, the poor signal intensity on MRI limits their clinical utility [8]. MNPs label is a major limitation for long-term tracking, as the magnetic resonance signal is lost over time due to cellular proliferation, especially with rapidly dividing cells. Hence, more efficient cellular-internalizing methods are highly preferable. Regardless of magnetic imaging, optical techniques offer improved high spatial resolution allowing the visualization of cell structures. They are used for the simultaneous visualization of multiple modalities with two or more fluorescent probes with different spectra, but have limited depth of imaging and poor absolute quantitative accuracy due to the absorption of light in tissues. Magnetic imaging has no practical limitation in terms of the depth of imaging; however, spatial resolution is poor and imaging with more than one probe is problematic [9]. Therefore, it is conceivable that one single agent to provide imaging in multiple imaging modalities (optical and magnetic) would be of great value to offer more comprehensive diagnostic information and the dynamics of disease progression. An example of such multifunctional NPs are FMNPs that bear two attractive features, fluorescence and superparamagnetism, allowing their intracellular movements to be controlled using magnetic force and monitored using a fluorescent microscopy, simultaneous delivery of fluorescence and magnetic particles to individual cells offers the opportunity of correlating optical images and MRI [8,10]. These features could lead to effective multifunctional drug-loaded MNPs that would facilitate increased drug transport rates, mucus penetration, antibiotic efficiency in biofilms and cellular imaging.

Semiconductor nanocrystals called quantum dots (QDs) is well known promising candidate as fluorescent materials for its high photostability, high emission quantum yield, narrow emission peak, size dependent wavelength tunability in comparison with organic dyes and fluorescent proteins. These properties of QDs make them more interesting for potential biomedical application such as protein trafficking, DNA detection, cellular imaging and dynamic studies of cell motility. QDs owing to their inorganic nature, are more robust than organic dyes and therefore their reduced photobleaching under light allows for real-time monitoring of biological events over extended periods of time [11,12]. Taking the advantages of QDs and MNPs, we designed, prepared and characterized new dual-marker particles, simultaneously combining both fluorescence nanocrystals, such as QDs and superparamagnetic nanoparticles (Fe_3_O_4_ NPs) in one entity. Considerable research has been devoted to the combination of magnetic and fluorescent properties in a single nanocomposites [1,2,13]. However, many of those synthetic processes usually involve complexed multi-step reactions [14-16]. Alternatively, a rapid one-pot self-assembly synthesis using controlled electrostatic force may improve photostability and colloidal stability. The cytotoxicity of prepared NPs was investigated in Vero cells using the trypan blue *in vitro* assay. The result indicates the cytotoxicity of FMNPs were reduced with dilution and allowed its potential use in biomedical application. The prepared FMNPs were further applied to cancer cell imaging using monoclonal antibodies against specific markers on cancer as a tool for therapy. Humanized monoclonal antibody CC49 (hCC49) [17] is a clinically validated antibody to target tumor-associated glycoprotein-72 (TAG-72) a well-known marker in colon carcinoma [18,19].

In this work, fragment antigen binding (Fab) region of hCC49 was conjugated with FMNPs, which was used for specific cancer cell imaging. Fluorescence microscopy showed significant preferential binding of the NPs conjugates by cells. Such a nanoprobe could potentially be used to image resections of cancer cells in real time and to correlate preoperative diagnostic images with intraoperative pathology at cellular-level resolution.

## Results and discussion

### Spectroscopic study of CdTe QDs and FMNPs

**Figure 1:**
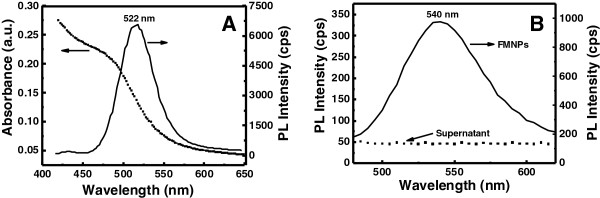
**Spectroscopic analysis of CdTe QDa and FMNPs. (A)** Absorbance and PL spectra of CdTe QDs; **(B)** PL spectra of FMNPs and supernatant solution.

The absorbance and photoluminescence (PL) intensity of QDs are shown in Figure 1A. The absorbance shoulder of QDs was located at 502 nm, while the PL peak of QDs was situated at 522 nm. According to Peng’s equation [20], the particle size of QDs was about 2.85 nm and concentration was 2.17 μM.

Fluorescence emission spectrum of FMNPs was recorded for the supernatant and residue after magnetic separation from the reaction mixtures. Residual solution remained strong fluorescence intensity while supernatant had ignorable PL intensity (Figure 1B). This indicates most of QDs capped on MNPs. Such a stable PL property is favorable for labeling cancer cells [8]. It is worth noting that the PL peak position (at 540 nm) for FMNPs in solutions red – shifted [8] compared with free QDs, suggesting that some free NPs may aggregate to form clusters through partial electrostatic force. The clustering of individual QDs causes slight degradation of energy level, leading to narrower energy band gap and band broadening. However, it did not observe any further uncontrolled aggregation of QDs that may bring fluorescence quenching in the experiments because of strong repulsion forces among QDs through their highly negative surface charges.

### Total internal reflection fluorescence (TIRF), transmission electron microscope (TEM) images and dynamic light scattering (DLS) studies of FMNPs

**Figure 2:**
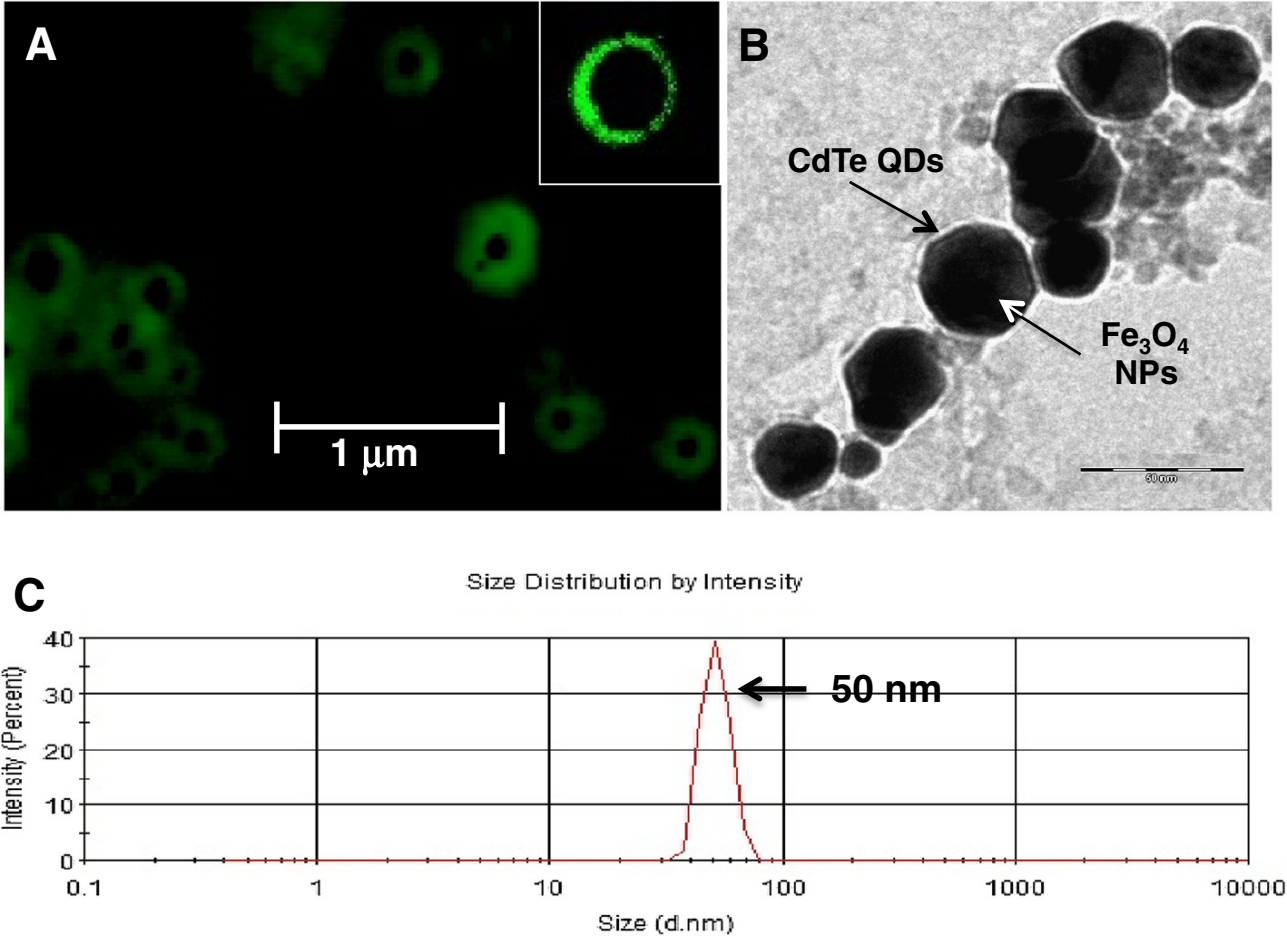
**Images of TIRF (A) and TEM (B), and DLS (C).** Bars in A and B denote 1 μm and 50 nm, respectively.

TIRF image shows the dark core made up of Fe_3_O_4_ NPs showing no fluorescence, whereas electrostatically adsorbed CdTe QDs covering the surface of NPs show distinct fluorescence (Figure 2A). Some single particle with district fluorescence property is also shown in inset of Figure 2A. TEM image of the FMNPs have shown the formation of core-shell NPs (Figure 2B). Fe_3_O_4_ NPs that are shown in black color, were covered by CdTe QDs that are shown transparent color. DLS data suggest that the size of FMNPs is about 50 nm (Figure 2C). Taking together these results smaller size CdTe QDs covered the surface of core Fe_3_O_4_ NPs. CTAB (not shown) between QDs and Fe_3_O_4_ NPs will avoid NPs to come in contact of each other, which would be helpful for better fluorescence emission. Due to the strong electrostatic interaction between the negatively charged -COO^-^ groups on the surface of the CdTe QDs and the positively charged quaternary amino groups of the CTAB, some QDs are forced to aggregate and forming a thick shell. It is better to notify that the size of NPs in TIRF image is bigger than TEM image. Usually, TIRF microscope has limitation to measure small particle size. In this study, TIRF microscope specially used to observe core (dark) and shell (green) structure of FMNPs.

### Superconducting quantum interference device (SQUID) study of FMNPs

**Figure 3:**
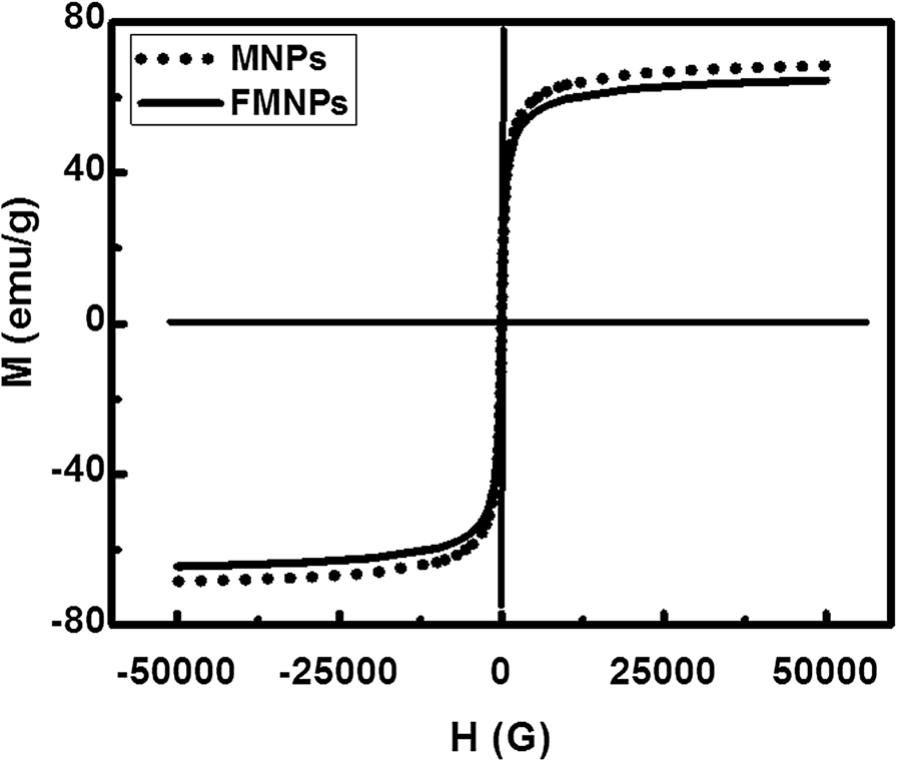
**Hysteresis loops of the FMNPs.** Magnetic properties of FMNPs and MNPs were measured using SQUID at 300 K.

The FMNPs were prepared by encapsulating inorganic magnetic particles covered by inorganic QDs. The MNPs were coated with CTAB and CdTe QDs using self-assembly method. Both the Fe_3_O_4_ MNPs and FMNPs exhibited typical superparamagnetic behavior due to no hysteresis. The remanence and coercivity are zero, illustrating that the particles respond magnetically to an external magnetic field, and redisperse rapidly when the magnetic field is removed. Figure 3 shows hysteresis loops of the proposed MNPs and FMNPs, which indicate that they possess a magnetic saturation value of about 71.0 and 65.0 emug^-1^. This large saturation magnetization makes them very susceptible to magnetic fields. The decrease in the overall magnetization values indicates that the Fe_3_O_4_ surface is covered with nonmagnetic materials such as mostly QDs and some organic materials of CTAB and stabilizers. These FMNPs have superparamagnetism, and no magnetism remains without the magnetic field. These properties make the FMNPs a useful tool for micro-separation in fluidic systems.

### Stability of FMNPs and cell viability in the presence of FMNPs

**Figure 4:**
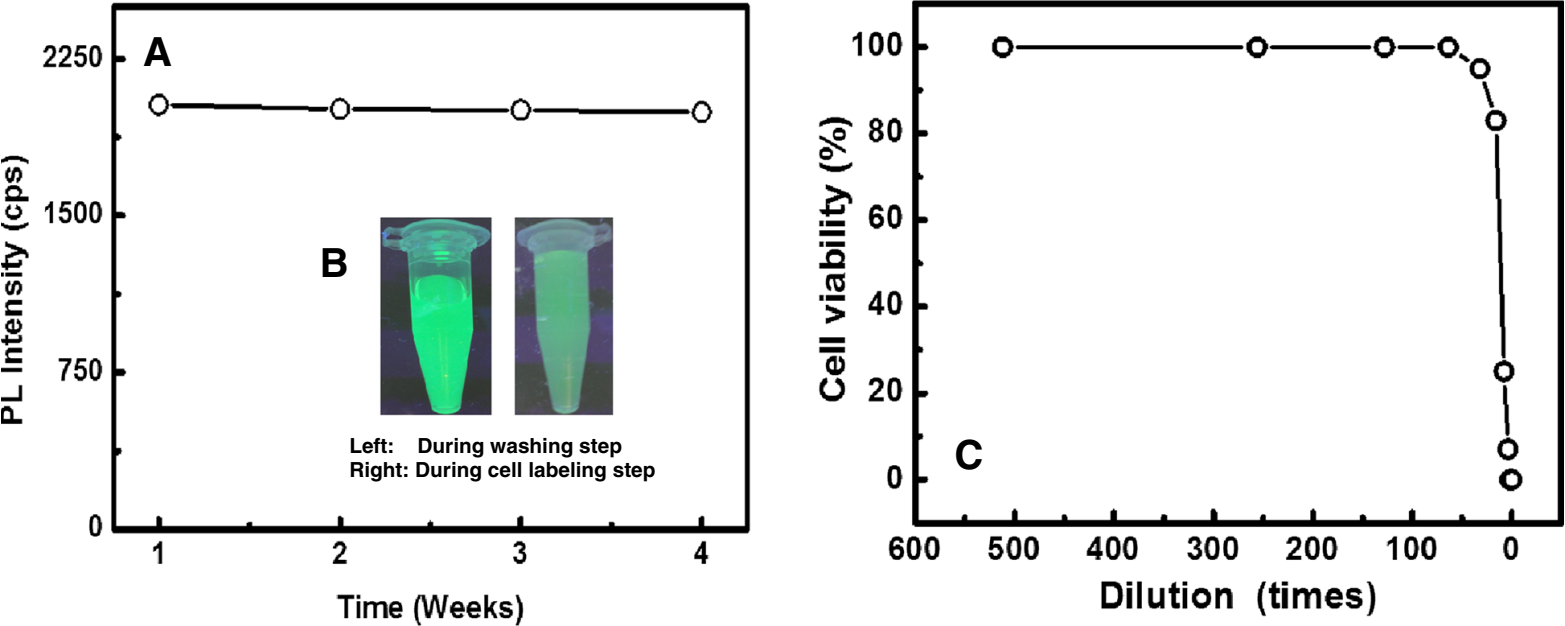
**Stability of prepared FMNPs at room temperature (A), fluorescence images of FMNPs at different stages (B), and cell viability in the presence of FMNPs (C).** The cytotoxicity of the FMNPs was measured using Vero cells by the trypan blue exclusion test.

As far as the stability of FMNPs, the FMNPs are in aqueous solution at room temperature for > 1 month. PL intensity of FMNPs did not deteriorate until 4 weeks (Figure 4A). This superior colloidal stability of FMNPs results from the cationic surfactant layer between QDs and MNPs which avoids QDs to come in close proximity to MNPs and retained its optical properties. FMNPs can be precipitate using external magnetic field and redispersed in water. This type of precipitation-redispersion can be repeated many times, suggesting that NPs structures are very robust in nature. Fluorescence images of FMNPs during washing and cell labeling are also shown stable optical properties (Figure 4B).

FMNPs are toxic for Vero cells, but dilution of FMNPs led to lower cytotoxicity. About 64 times diluted solution of FMNPs has shown no cytotoxicity (Figure 4C). Compared with TGA-capped CdTe QDs, the as-prepared QDs showed lower cytotoxicity under the same conditions (data not shown). Generally, CdTe QDs are highly toxic for cells due to the release of Cd^2+^ ions [21]. However, the prepared FMNPs reduce the cytotoxicity of QDs to a small extent. A reasonable interpretation is that the outer surface of FMNPs was covered by quaternary ammonium groups and leading to less released Cd^2+^ ions.

### Confirmation of FMNPs conjugated with Fab region of hCC49 antibody

**Figure 5:**
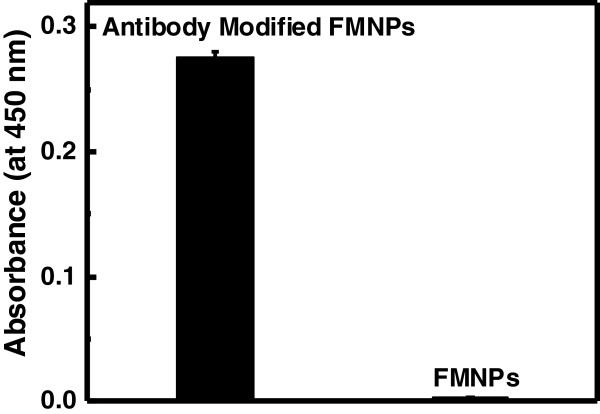
**ELISA result for antibody conjugated with FMNPs.** Anti-FLAG antibody and hCC49 antibody Fab region conjugation were performed on FMNPs.

FMNPs conjugated with Fab region of hCC49 antibodies are confirmed with bead enzyme-linked immunosorbent assay (ELISA). Higher signal was observed with Fab antibody modified FMNPs than only FMNPs (Figure 5), suggesting that the FMNPs was successfully conjugated with antibodies.

### Cellular imaging of colon carcinoma cells using FMNPs conjugated with Fab region of hCC49 antibody

**Figure 6:**
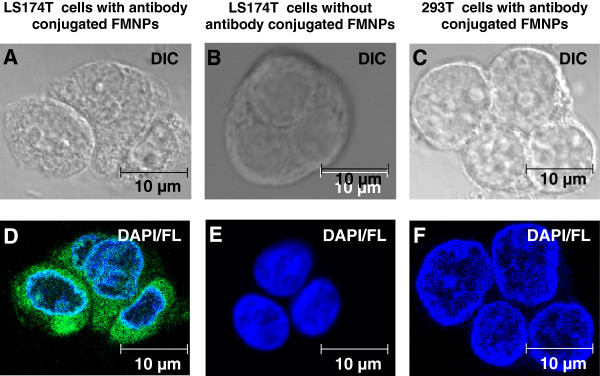
**Confocal laser scanning microscopy images of cancer cells.** Differential interference contrast (DIC) images of LS174T cells with **(A)**, without **(B)** hCC49 antibody Fab region conjugated FMNPs, and 293 T cells with hCC49 antibody Fab region conjugated FMNPs **(C)**. Merged images of DAPI/Fluorescence for LS174T cells with **(D)**, without **(E)** hCC49 antibody Fab region conjugated FMNPs, and 293 T cells with hCC49 antibody Fab region conjugated FMNPs **(F)**.

Specific binding between FMNPs and cancer cells was observed by confocal laser scanning microscopy *in vitro*. When LS174T cancer cells were incubated with FMNPs conjugated with Fab region of hCC49 antibodies, green fluorescence around the nucleus (Figure 6D) confirming Fab region of antibodies bound with sialylated sugar chain in TAG-72 region of LS174T cancer cells. As a negative control, LS174T cancer cells were incubated without hCC49 antibodies Fab region-conjugated FMNPs. However, negative cells show only nucleus staining by 4′,6-diamidino-2-phenyindole (DAPI) (Figure 6E), no green fluorescence around the nucleus. For more specificity, HEK293 cells were incubated with FMNPs conjugated with Fab region of hCC49 antibodies and merged image of DAPI and fluorescence confirmed no green fluorescence around the nucleus (Figure 6C and F). This indicates that HEK293 cells have no specific binding site of Fab region of hCC49 antibodies. Cell morphology was maintained after being incubated with the FMNPs, which implies that the nanocomposites had no cytotoxic effect on the cancer cells. In addition, these confocal images confirmed that the NPs were bound the cell surface and the NPs accumulated uniformly around cell surface.

## Conclusion

In conclusion, dual modal fluorescent-magnetic nanocomposites based on fluorescent CdTe QDs and Fe_3_O_4_ MNPs were developed by a simple LbL fabrication. This self-assembly technique retains the magnetic and fluorescence properties of FMNPs. FMNPs had good optical properties, and decent saturation magnetization. Further *in vitro* studies showing less toxicity at 64 fold dilutions and the ability to interact with cancer cells showing the ability of the FMNPs probes for imaging. Ultimately, it is believed that these particles will provide a new class of multimodal NPs for the complex biologic systems.

## Methods

### Materials

Cadmium perchlorate hydrate, thioglycolic acid solution (TGA) and hexadecyltrimethyl ammonium bromide (CTAB) were purchased from Sigma-Aldrich (St. Louis, Missouri, USA). Aluminum telluride (Al_2_Te_3_) with the highest purity was acquired from Cerac Inc. (Albuquerque, NM, USA). Ferric chloride, ferrous sulfate, trisodium citrate dihydrate and ammonium solution were purchased from Wako Pure Chem. Inc. (Osaka, Japan). LS174T human colon adenocarcinoma cell line (ATCC No. 50845) and Vero cell (No. CCL-81) strains were purchased from ATCC (Rockville, MD, USA).

### Synthesis of CdTe QDs

CdTe-QDs has been synthesized as previously reported [22]. Shortly, 0.985 g (2.35 mmol) of Cd(ClO_4_)_2_ · 6H_2_O was dissolved in 125 ml of water, and 5.7 mmol of the TGA added under stirring, followed by adjusting pH to appropriate values (11.4–11.6) by dropwise addition of 1 M NaOH. The solution was placed in a three-necked flask and deaerated by nitrogen gas-bubbling for 30 min. Under stirring, H_2_Te gas (generated by the reaction of 0.2 g (0.46 mmol) of Al_2_Te_3_ lumps with 15–20 ml of 0.5 M H_2_SO_4_ under nitrogen atmosphere) was passed through the solution together with a slow nitrogen flow for 20 min. CdTe precursors formed were converted to CdTe nanocrystals by refluxing the reaction mixture at 100°C under open-air conditions with condenser attached for 20 min. The UV and PL spectra of CdTe QDs were recorded using a Tecan infinite M 200 spectrophotometer. The samples were excited at 380 nm, and the exciting and the emission slits were 5 and 10 nm, respectively.

### Synthesis of Fe_3_O_4_ NPs

The preparation of citrate-capped Fe_3_O_4_ NPs was synthesized using the previously described method with minor modification [23]. The reaction mixture contained 1.622 g of FeCl_3_ · 6H_2_O and 0.994 g of FeCl_2_ · 4H_2_O, were mixed with 40 ml of distilled water. Five ml of concentrated ammonia aqueous (28%) was added in this solution and heated at 90°C. Then, 4.4 g sodium citrate was added and continued the reaction for 30 min. The MNPs thus obtained were washed with anhydrous ethanol and redispersed in deionized water for further use. Particle size and electric charge the prepared Fe_3_O_4_ NPs were measured by dynamic light scattering (ZetaSizer Nano-ZS, Malvern, UK).

### Preparation of FMNPs

New synthesis method for fluorescent-magnetic nanocomposites structure were prepared based on simple and efficient LbL self-assembly method [24]. Briefly, 100 ml Fe_3_O_4_ NPs were dispersed in 5 ml of CTAB (10 mg/ml) solution under vagarious stirring. Next, 200 ml of ammonia solution was added and the mixture was stirred at room temperature for 30 min. Cationic surfactant CTAB capped Fe_3_O_4_ NPs by electrostatic force at this stage. Then, 200 ml of CdTe QDs was added and mixtures were stirred at room temperature for 2 h for binding of QDs with CTAB. The resulting FMNPs were collected by external magnetic field, washed three times with MilliQ water and redispersed in MilliQ water for further use. The PL spectra of FMNPs were recorded using a Tecan infinite M 200 spectrophotometer. The samples were excited at 380 nm, and the exciting slit and the emission slit were 5 and 10 nm, respectively.

### TIRF microscope and TEM Imaging of FMNPs

TIRF microscope (Leica AM TIRF MC, Germany) was used to observe fluorescence image of FMNPs. Two hundred microliter FMNPs solutions were loaded in glass-bottom-dishes and samples were excited using 400 nm wavelength. The TEM image of FMNPs was recorded using JEM 2000FX II-TEM (JOEL Ltd., Akishima, Japan). The samples were spotted onto carbon grids (Okenshoji, Tokyo, Japan), dried at room temperature and observed at 50,000 × magnification operating at 160 kV.

### Superconducting quantum interference device (SQUID) measurement

SQUID magnetometry was used to measure the saturation magnetizations of naked MNPs and FMNPs at 300 K. Aliquots (200 μl) of the MilliQ suspension of MNPs and FMNPs were placed in micro centrifuge tube and allowed to vacuum evaporation (Centrifugal evaporator CVE-2000, EYELA, Japan). Magnetic measurements were made on a Quantum Design MPMS-7 SQUID (San Diego, CA, USA) magnetometer.

### Cell lines and cell cultures

HEK293 was obtained from Riken Bio Resource Center (RCB1637). LS174T cells were cultured in 60 mm culture plates (TPP, Trasadingen, Switzerland) with minimal essential media-eagle (Sigma-Aldrich, Tokyo, Japan) containing 10%(v/v) fetal bovine serum (Invitrogen, San Diego, CA, USA), supplemented with 1%(v/v) antibiotic solution containing penicillin, streptomycin, fungizone (Sigma-Aldrich, Tokyo, Japan) and incubated at 37°C in 5% CO_2_ incubator (MCO-175 Sanyo, Osaka, Japan).

HEK293 cells were cultured in 60 mm culture plates with MEM/EBSS (HyClone Laboratories Inc., Utah, USA) containing 2 mM L-glutamine, 1% non-essential amino acid (Invitrogen, Carlsbad, CA, USA), 10% fetal bovine serum, supplemented with 1% (v/v) antibiotic solution containing penicillin, streptomycin, fungizone and incubated at 37°C in 5% CO_2_ incubator. Both the cell lines were grown till confluence and splitting was done in 1:5 ratio once every week by trypsinisation using TrypLE Express (Life Technologies Japan LTD., Minato-Ku, Tokyo, Japan) for 15 min at 37°C in 5% CO_2_ incubator.

### Trypan blue test

The cytotoxicity of the FMNPs were determined using the trypan blue exclusion test, which determines the number of viable cells present in a cell suspension. In 96-well plates, 100 μl suspension of Vero cells (5 × 10^4^ cells/ml) in 5%(v/v) horse serum containing minimum essential medium was added per well and incubated in a 5% CO_2_ humidified incubator at 37°C for 24 h. Then different diluted solutions of FMNPs were added to each well and further incubate at 37°C for 24 h. After medium was removed, 0.25% trypsin was added in all wells. The plate was centrifuged (Centrifuge 5430, Eppendorf AG, Germany), removed supernatant and redispersed in 5%(v/v) horse serum minimal essential medium. The 96-well plates were further incubated for 15 min. Fifteen microliter of trypan blue was added to each cell well. The resulting mixture was gently shaken for 10 min at room temperature. Cell numbers were counted using haemocytometer (Line Seiki, Tokyo, Japan) under microscope (Model CHT, Olympus optical Co. Ltd., Tokyo, Japan) and the percentage of viability was calculated.

### Preparation of hCC49 antibody Fab regions

The gene of hCC49 antibody [19] was amplified from plasmid pDong1 (hCC49), which is a gift from Professor Hiroshi Ueda of Chemical Resources Laboratory of Tokyo Institute of Technology by PCR using primers bx-FLAG-hCC49VHCH-F (5’-CACCATGAAGATACTCCTTGCTATTGCATTAATGTTGTCAACAGTAATGTGGGTGTCAACATGGAGCCACCCGCAGTTCGAAAAGATGAAATACCTATTG-3’) and hCC49VHCH-R (5’-CTATGCGGCCCCATTCAG-3’) for hCC49VHCH1, primers bx-FLAG-hCC49VLCL-F (5’-CACCATGAAGATACTCCTTGCTATTGCATTAATGTTGTCAACAGTAATGTGGGTGTCAACAGACTACAAGGATGACGATGACAAGGATATTGTGATGACC-3’) and hCC49VLCL-R (5’-TCACTCTCCCCTGTTGAA-3’) for hCC49VLCL. The amplified genes were inserted into pENTR/D-TOPO (Invitrogen), respectively, to make pENTR/D-hCC49VHCH1 and pENTR/D-hCC49VLCL by the topoisomerase reaction. Using the above plasmids, the gene was inserted into pDEST8 using Gateway technology (Invitrogen) to construct pDEST-hCC49VHCH1 and pDEST-hCC49VLCL, which were then used to transform *E. coli* BmDH10Bac to obtain the recombinant *Bombyx mori* nucleopolyhedrin (BmNPV) bacmids, rBmNPV-hCC49VHCH1 and rBmNPV-hCC49VLCL. The recombinant bacmids (10 μg each) were mixed with one-tenth volume of DMRIE-C (Invitrogen) and incubated at room temperature for over 45 min. Fifty microliters of this mixture was injected into a silkworm larva on the first day of the fifth instar larvae (Ehime Sansyu Co. Ltd., Ehime, Japan). Injected silkworm larvae were reared for 5–7 days, and the hemolymph collected was centrifuged to remove hemocytes at 2400 × g for 10 min at 4°C. The supernatant was used as a hemolymph sample for purification. Fab antibodies were purified with an ANTI-FLAG M2 Affinity Gel (Sigma) according to the instructions provided by the manufacturer.

### Conjugation of hCC49 antibody Fab region with FMNPs and its binding activity

To apply FMNPs on bioimaging, the NPs were conjugated with hCC49 antibodies Fab region to assess their potential as fluorescent probes for LS174T cancer cells. Fab region of hCC49 antibodies has binding affinity with sialylated sugar chain in TAG-72 region of LS174T cancer cells. Electrostatic interaction between positively charged FMNPs (+18.3 mV, measured by ZetaSizer, Nano-ZS, Malvern, UK) and negatively charged of hCC49 antibodies (−3.53 mV, Tris glycin at pH 8.5, measured by ZetaSizer, Nano-ZS, Malvern, UK) was used to bind each other. NPs were dissolved in hCC49 antibodies (final concentration of 20 μg/ml) and kept at 10°C for 2 h with continuous stirring. To check whether antibodies were bound with FMNPs or not, at first FMNPs were blocked with 2% bovine serum albumin (BSA). After incubation at room temperature for 2 h, samples were washed three times with PBS buffer solution and redispersed in anti-FLAG antibodies conjugated horseradish peroxidase (Sigma, USA) with a final concentration of 1 ng/ml. After incubation at room temperature for 1 h, samples were washed three times with PBS buffer solution and transferred to a new tube. Signal was developed with 100 μl 3,3’,5.5’-tetramethylbenzidine (TMBZ, Sigma) substrate solution (20 μg ml^-1^ TMBZ and 0.2 μl H_2_O_2_ in 1 ml NaOAc, pH 6.0) for 5–30 min at 25°C. The reaction was stopped by adding 100 μl of 10% H_2_SO_4_ and absorbance of reaction supernatant was recorded at 450 nm with a reference at 655 nm using a micro plate reader (Model 680, Bio-Rad, Hercules, CA, USA).

### Cellular imaging using hCC49 antibody Fab region conjugated FMNPs

Colon carcinoma cancer cells (LS174T) were seeded on slide glass 24 h prior to labeling and staining. LS174T cells were cultured with hCC49-conjugated NPs, without hCC49-conjugated NPs and HEK293 cells were cultured with hCC49-conjugated NPs for 24 h in a 37°C humidified incubator maintained at 5% CO_2_. After incubation, fixation of the cells were carried out using 10%(v/v) formalin for 20 min and rinsed four times using PBS (pH 7.5). Then, 50 mmol NH_4_Cl solutions were added and washed four times with PBS (pH 7.5). Next, 4% BSA solution was used as a blocking agent for 1 h at RT. Following labeling, the slide glasses were washed four times PBS buffer (pH 7.5). After washes, cellular nuclei were stained with 1% (v/v) solution of DAPI in 2% BSA solution in PBS buffer (pH 7.5) for 1 h and washed with PBS (pH 7.5) four times. Confocal images were acquired using a confocal laser scanning microscope (LSM 700, Carl Zeiss Microimaging GmbH, 07740 Jena, Germany) and Image processing was performed using LSM Software ZEN 2010.

## Abbreviations

BSA: Bovine serum albumin; CTAB: Hexadecyltrimethyl ammonium bromide; CdTe QD: Cadmium telluride quantum dot; DAPI: 4′,6-Diamidino-2-phenyindole; DLS: Dynamic light scattering; ELISA: Enzyme-linked immunosorbent assay; Fab: Fragment antigen binding; Fe3O4 NP: Iron oxide nanoparticle; FMNP: Fluorescent-magnetic nanoparticle; hCC49: Humanized monoclonal antibody CC49; HEK: Human embryo kidney; LbL: Layer by Layer; MNP: Magnetic nanoparticle; MRI: Magnetic resonance imaging; NP: Nanoparticle; PL: Photoluminiscence; QD: Quantum dot; SQUID: Superconducting quantum interference device; TAG-72: Tumor associated glycoprotein-72; TEM: Transmission electron microscopy; TGA: Thioglycolic acid; TIRF: Total Internal Reflection Fluorescence; TMBZ: 3,3’,5.5’-tetramethylbenzidine.

## Competing interests

The authors declare that they have no competing interests.

## Authors’ contributions

SRA performed this experiment in all state and also wrote this manuscript; JD carefully observed in all stages both experimental and manuscript writing; MY performed cellular imaging study; TK took TEM image and gave direction for biocompatibility test; JBL and EYP were principal supervisor in this research and gave us valuable guidance to improve this work. All authors read and approved the final manuscript.
